# Reversibility and energy dissipation in adiabatic superconductor logic

**DOI:** 10.1038/s41598-017-00089-9

**Published:** 2017-03-06

**Authors:** Naoki Takeuchi, Yuki Yamanashi, Nobuyuki Yoshikawa

**Affiliations:** 1 Institute of Advanced Sciences, Yokohama National University, 79-5 Tokiwadai, Hodogaya, Yokohama 240-8501 Japan; 2 PRESTO, Japan Science and Technology Agency, 4-1-8 Honcho, Kawaguchi, Saitama 332-0012 Japan; 3 Department of Electrical and Computer Engineering, Yokohama National University, 79-5 Tokiwadai, Hodogaya, Yokohama 240-8501 Japan

## Abstract

Reversible computing is considered to be a key technology to achieve an extremely high energy efficiency in future computers. In this study, we investigated the relationship between reversibility and energy dissipation in adiabatic superconductor logic. We analyzed the evolution of phase differences of Josephson junctions in the reversible quantum-flux-parametron (RQFP) gate and confirmed that the phase differences can change time reversibly, which indicates that the RQFP gate is physically, as well as logically, reversible. We calculated energy dissipation required for the RQFP gate to perform a logic operation and numerically demonstrated that the energy dissipation can fall below the thermal limit, or the Landauer bound, by lowering operation frequencies. We also investigated the 1-bit-erasure gate as a logically irreversible gate and the quasi-RQFP gate as a physically irreversible gate. We calculated the energy dissipation of these irreversible gates and showed that the energy dissipation of these gate is dominated by non-adiabatic state changes, which are induced by unwanted interactions between gates due to logical or physical irreversibility. Our results show that, in reversible computing using adiabatic superconductor logic, logical and physical reversibility are required to achieve energy dissipation smaller than the Landauer bound without non-adiabatic processes caused by gate interactions.

## Introduction

The energy efficiency of a computer has been improving by reducing the physical size of complementary metal–oxide–semiconductor (CMOS) logic devices. It is estimated that the switching energy of a single CMOS gate is approximately 1000*k*_B_ *T* for the modern device size and will reach 100*k*_B_ *T* for a sub-5-nm gate length^1^, where *k*_B_ is the Boltzmann constant and *T* is temperature. Note that, in practical use, static power consumption generated by leakage currents and dynamic energy dissipation required to charge and discharge wires push up the average of energy dissipation of a single gate^2^. In a non-adiabatic device such as CMOS, the minimum switching energy is expected to be approximately 100*k*_B_ *T*, because the switching energy corresponds to the height of energy barrier^3^, which needs to be much larger than *k*_B_ *T* to define two distinguishable logic states. Therefore, the reduction in physical size will no longer help improve energy efficiency in future computers. In order to achieve a switching energy even smaller than 100*k*_B_ *T*, reversible computing^4^ is attracting attention. In reversible computing, logical entropy, which is given as Shannon entropy^5^ regarding binary switches^6^, is conserved and therefore energy dissipation required for a logic operation can be even smaller than the thermal limit given by *k*_B_ *T *ln2, or the Landauer bound^7^. Several types of reversible logic devices have been proposed so far, that include adiabatic CMOS^8^, nanomagnetic logic^9, 10^, nanomechanical devies^11^, and superconductors^12^.

In a previous study, we proposed a reversible quantum-flux-parametron (RQFP) as a reversible superconductor logic gate^13^. We numerically demonstrated that the energy dissipation required for a logic operation using an RQFP gate can be arbitrarily decreased by lowering operation frequencies. This comes from the fact that the RQFP gate is physically, as well as logically, reversible, as will be shown later. On the other hand, it is predicted that, in irreversible logic gates, the energy dissipation during a logic operation exceeds Landauer bound^7^ because of the reduction in logical entropy. However, the physical mechanism of how energy is dissipated during an irreversible logic operation has been unclear. In this study, we reveal the mechanism of the energy dissipation in irreversible logic gates using numerical calculation. We first show that the RQFP gate is physically reversible by showing the time evolution of the phase differences of Josephson junctions in the RQFP gate^14^. By way of comparison, we show the time evolution and energy dissipation of logically or physically irreversible gates. Taking into account the above results, we discuss how the energy greater than the Landauer bound is dissipated in irreversible logic gates, and why the energy dissipation can be arbitrarily reduced in the RQFP gate. The obtained results will help understand the relationship between reversibility and energy dissipation and could move the discussion on limits of computing from the theoretical stage to the physical stage.

## Results and Discussion

### Reversible Quantum-Flux-Parametron

**Figure 1:**
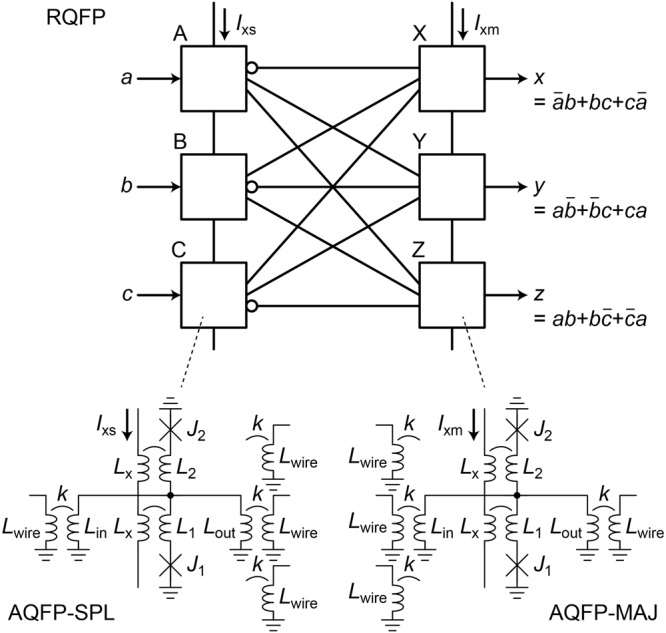
Schematic of the RQFP gate. The gate is composed of six AQFP gates, which are represented by the white boxes. The three gates (**A–C**) work as three-output SPL gates and the others (**X–Z**) work as three-input MAJ gates. *a*, *b*, *c* are input data and *x*, *y*, *z* are output data. The RQFP gate is logically reversible and symmetrical in terms of circuit schematic. *J*_1_ = *J*_2_ = 50 μA, *L*_1_ = *L*_2_ = 1.32 pH, *L*_in_ = *L*_out_ = *L*_wire_ = 10.5 pH, *k* = 0.4. The Josephson junctions are underdamped without shunt resistors. Invert functions were achieved by changing the polarity of the coupling coefficient, *k*.

Figure 1 shows the schematic of the RQFP gate, which is composed of six adiabatic quantum-flux-parametron (AQFP) gates^15, 16^. AQFP is an adiabatic superconductor logic based on the quantum-flux-parametron (QFP)^17^ proposed by Eiichi Goto. A single AQFP gate can change its logic state adiabatically^16^, while non-adiabatic processes can occur in complex circuits depending on how we combine AQFP gates, as will be shown later. The white boxes correspond to AQFP gates, the circuit parameters of which are similar to those in a previous work^16^ and are shown in the caption. The critical currents of the Josephson junctions are 50 μA, and device parameters such as sub-gap resistance are based on the AIST advanced process (ADP2)^18^. The gates labeled as A, B, and, C work as three-output splitter (SPL) gates and the others labeled as X, Y, and, Z work as three-input majority (MAJ) gates, the operation principles of which are described in the literature^13^. The SPL gate is a multi-fanout buffer gate and the MAJ gate is a logic gate, whose logic state is determined by the majority vote of inputs. *I*_xs_ and *I*_xm_ are the excitation currents for the SPL gates and MAJ gates, respectively. When excitation currents are applied, either *J*_1_ or *J*_2_ in the AQFP gate switches and the output current is generated on the inductor, *L*_out_. Since the schematics of the SPL and MAJ gates are the same, where the direction of data determines the logic functions of the gates, the RQFP gate is totally symmetrical and data can propagate bi-directionally. In Fig. 1, *I*_xs_ is activated first, so that data propagate from the input ports, *a*, *b*, *c*, to the output ports, *x*, *y*, *z*. If *I*_xm_ is activated first, data propagate in the opposite direction with the same logic operations. The obtained logic operations are shown in the figure. From the truth table^13^, it is clear that the RQFP gate is logically reversible, where input and output data combinations are bijective, i.e., the input data can be always predicted from output data and logical entropy is conserved.

**Figure 2:**
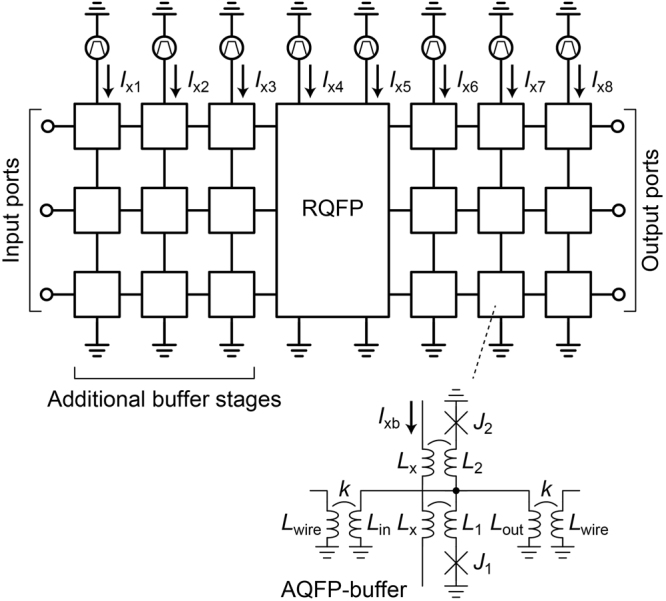
Schematic of the circuit used in simulation. Before and after the RQFP gate, three additional buffer stages are placed so as to avoid interactions from the input and output ports. *I*_x1_ through *I*_x8_ are excitation currents, where the RQFP gate is activated using *I*_x4_ and *I*_x5_. The circuit parameters of the buffer gate are the same as those shown in Fig. 1. Input currents of −50 μA and 50 μA are applied to the input ports as logic 0 s and 1 s, respectively.

**Figure 3:**
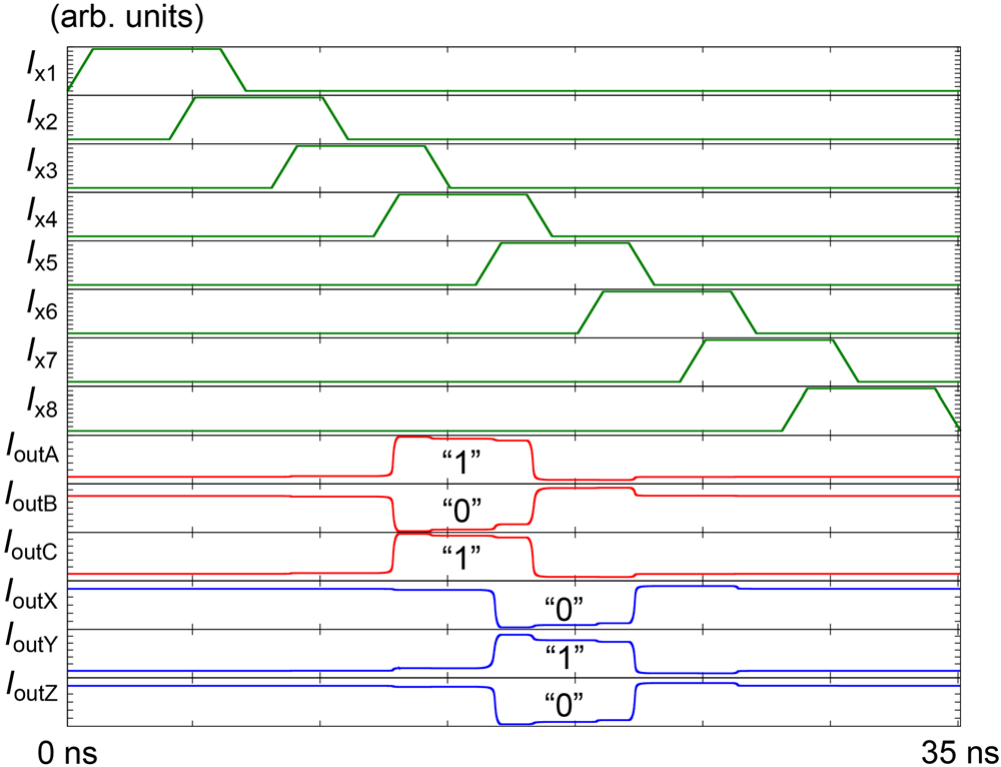
Transient analysis results of the RQFP gate. When *I*_x4_ is activated, the SPL gates in the RQFP gate are excited and generate output currents (*I*_outA_, *I*_outB_, *I*_outC_). When *I*_x5_ is activated, the MAJ gates in the RQFP gate are excited and generate output currents (*I*_outX_, *I*_outY_, *I*_outZ_). As excitation currents are activated in turn, three-bit data propagate from the input ports towards the output ports.

Figure 2 shows the circuit used for numerical calculation in this study. After and before the RQFP gate, three buffer stages are added, because energy interactions near the first and the last gates are complicated^19^. Input currents of −50 μA and 50 μA were added to the input ports, so as to generate logic 0 s and 1 s, respectively. Figure 3 shows the transient analysis results of the circuit represented in Fig. 2, where JSIM_n^20^ is used for the simulation and the rise and fall time of excitation currents is 1,000 ps. *I*_x1_ to *I*_x8_ are the excitation currents for each excitation stage, *I*_outA_ to *I*_outC_ are the output currents of the SPL gates in the RQFP gate, and *I*_outX_ to *I*_outZ_ are the output currents of the MAJ gates in the RQFP gate. As excitation currents are activated in turn, data propagate from the first stage toward the last stage. When *I*_x4_ and *I*_x5_ are activated, SPL and MAJ gates generate output currents, respectively.

**Figure 4:**
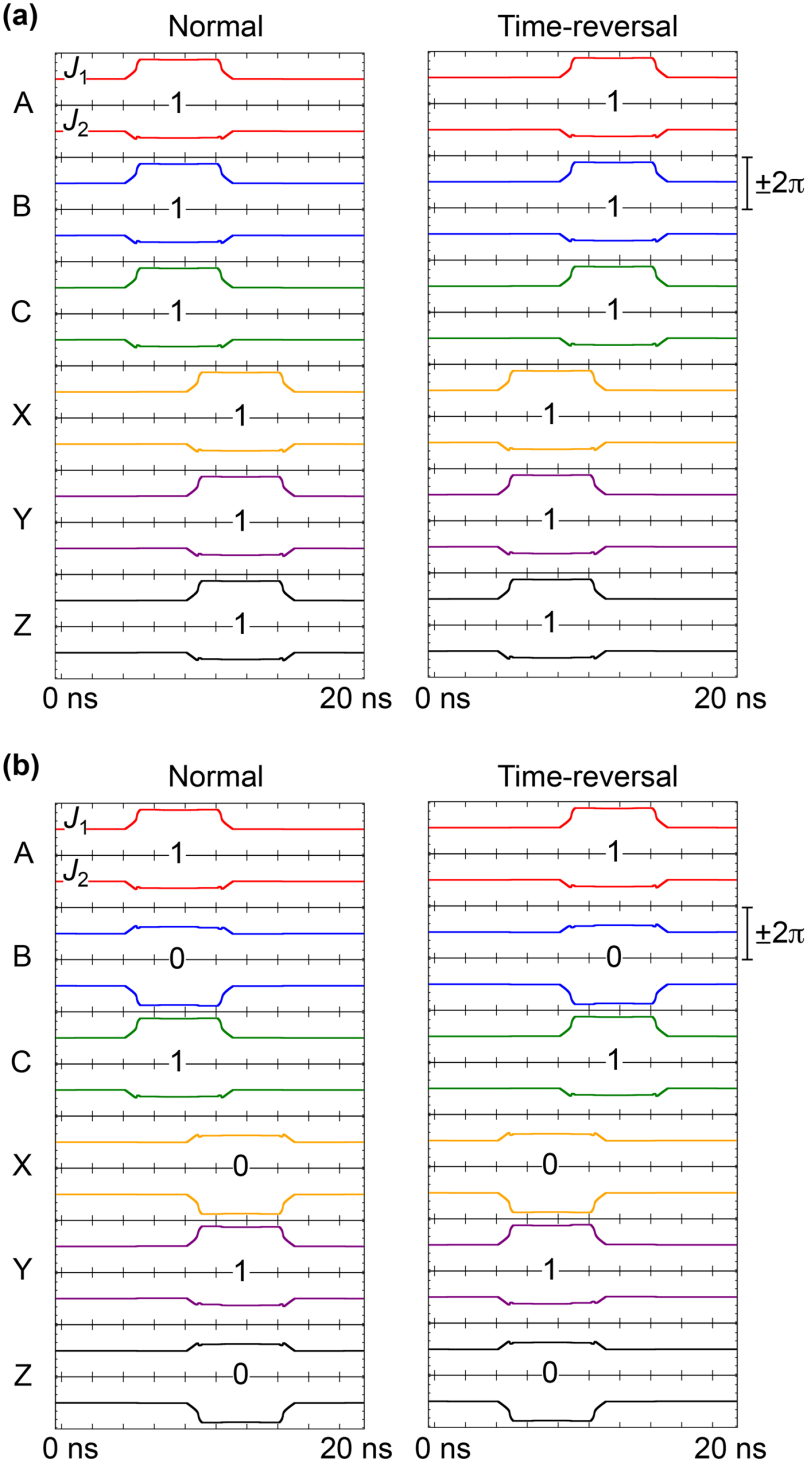
Evolution of phase differences of Josephson junctions in the RQFP gate. (**a**) *a* = 1, *b* = 1, *c* = 1. (**b**) *a* = 1, *b* = 0, *c* = 1. The phase differences change time reversibly for all the input data combinations.

In AQFP logic, the directions of currents depend on logic states but internal energy is the same between the logic states 0 and 1. Also, the combination of input data for the RQFP gate is limited to the following two patterns; all of the inputs (*a*, *b*, *c*) are in the same logic state, or one of the inputs is different from the others. Therefore, here we treat only the following two data combinations: (*a*, *b*, *c*) = (1, 1, 1) and (*a*, *b*, *c*) = (1, 0, 1). In order to investigate the physical reversibility, we analyze the time evolution of the phase differences of Josephson junctions, which are state variables in the RQFP gates, for both normal and time-reversal processes^14^ because the currents, voltages, and internal energy in Josephson circuits are given by the phase differences. In the time-reversal process, input currents are given to the output ports and the excitation currents are activated in the order from *I*_x8_ to *I*_x1_, i.e., in the opposite order from that shown in Fig. 3. Figure 4 shows the evolution of phase differences of the Josephson junctions in the RQFP gate for both normal and time-reversal processes, in which the labels (A to Z) identify the AQFP gates shown in Fig. 1. Each AQFP gate includes a pair of Josephson junctions, *J*_1_ and *J*_2_, as shown in Fig. 1, where *J*_1_ switches for logic 1 and *J*_2_ switches for logic 0. For all the input data combinations, the logic states of the AQFP gates are the same between normal and time-reversal processes, and the evolution of the phase differences is totally symmetrical about time, or time reversible. Therefore, the RQFP gate is physically reversible.

**Figure 5:**
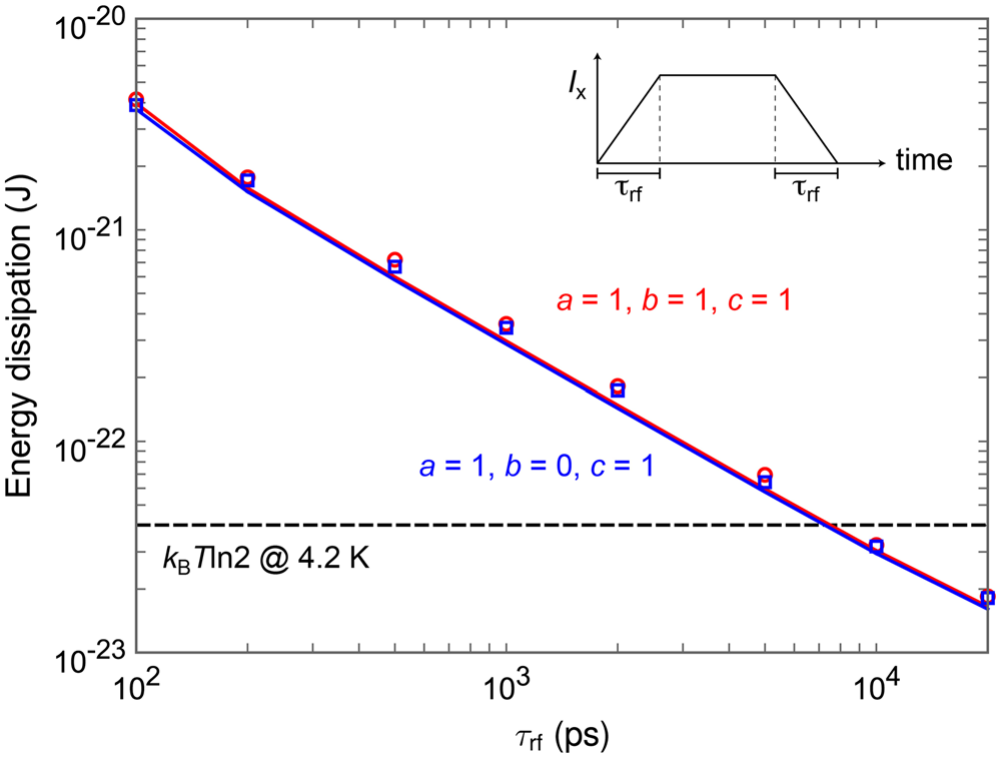
Simulation results of the energy dissipation per logic operation of the RQFP gate as a function of the rise and fall time of excitation currents, τ_rf_. The lines show the results without taking into account thermal noise, and the markers show the averaged results over 500 iterations with thermal noise at 4.2 K. As τ_rf_ increases, energy dissipation reduces linearly for all the input data combinations.

Figure 5 shows the energy dissipation per logic operation of the RQFP gate as a function of the rise and fall time of excitation currents, τ_rf_. The energy dissipation was calculated by integrating excitation currents and the voltages across the excitation inductor, *L*_x_, over time^21^. The lines show the calculation results without taking into account thermal noise. The markers show the averaged values over 500 iterations with 4.2 K thermal noise, where thermal noise current sources are added to Josephson junctions in parallel. The amplitude of the thermal noise currents is given using the Monte Carlo method and follows the Gaussian law with the standard deviation given by (2*k*_B_ *T*/*R*Δ*t*)^0.5^, where Δ*t* is a simulation time step and *R* is the sub-gap resistance^22^. In this study, Δ*t* = 0.2 ps and *R* = 200 ohm. As shown in a previous study^16^, it is confirmed that the average of energy dissipation at 4.2 K corresponds to that at 0 K. For all the input data combinations, energy dissipation reduces linearly as τ_rf_ increases, and the energy dissipation falls even below the Landauer bound for τ_rf_ of approximately 7,000 ps. This is because, due to the physical reversibility, the switching events of Josephson junctions approach quasi-static adiabatic processes as the potential energy is changed more slowly. It should be noted that, for τ_rf_ = 20,000 ps, the energy dissipation of each AQFP gate included in the RQFP gate is only approximately 3 × 10^−24^ J.

### Logical Reversibility

**Figure 6:**
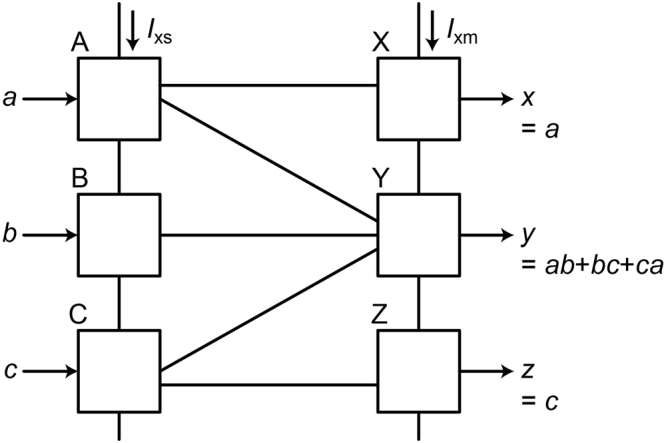
Schematic of the 1-bit-erasure gate, which is composed of six AQFP gates. The input data, *a* and *c*, are copied to the output data, *x* and *z*. The output data, *y*, takes the majority vote of the input data, *a*, *b*, *c*. Therefore, the input data, *b*, is erased, i.e., the 1-bit-erasure gate is logically irreversible.

**Figure 7:**
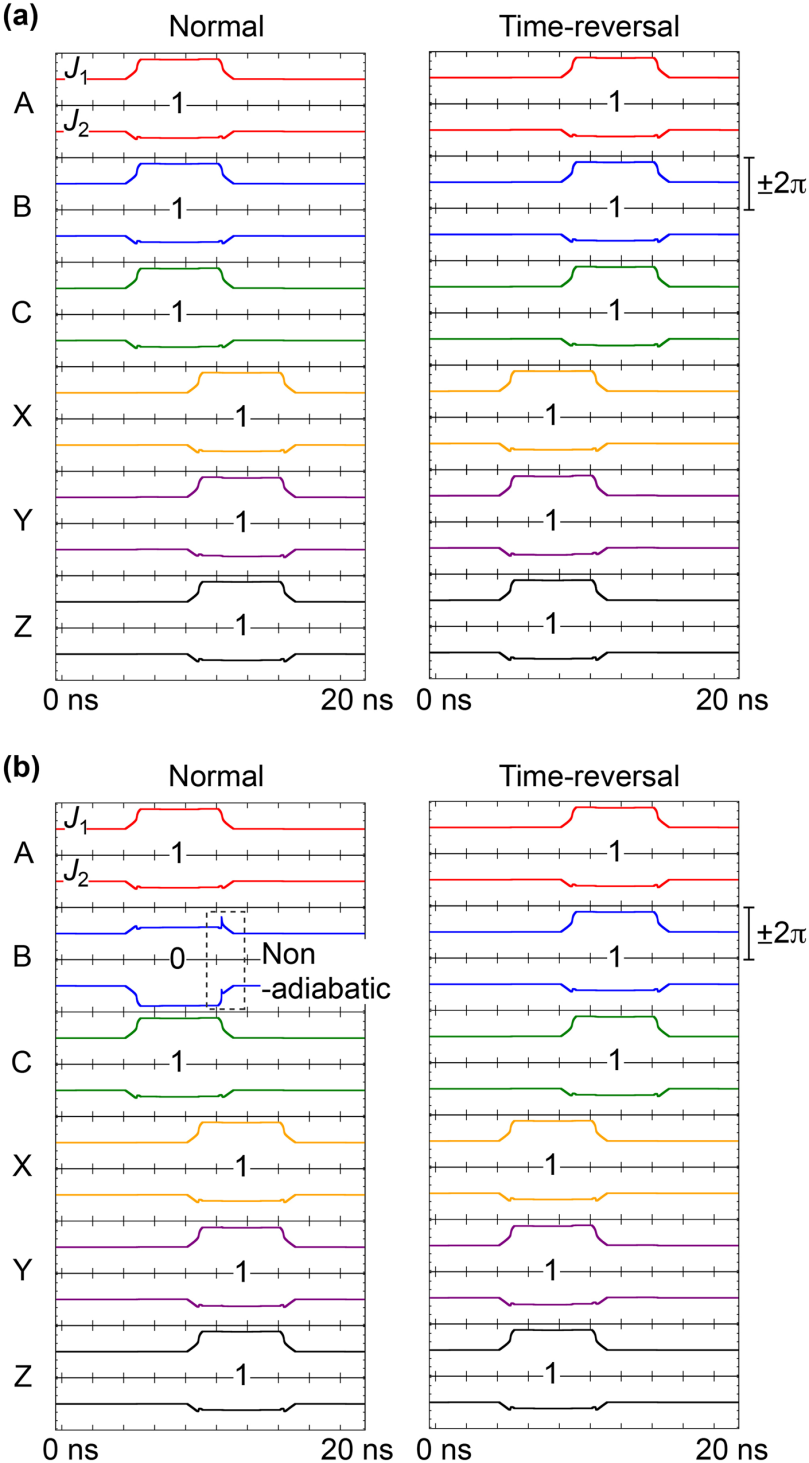
Evolution of phase differences of Josephson junctions in the 1-bit-erasure gate. (**a**) *a* = 1, *b* = 1, *c* = 1. (**b**) *a* = 1, *b* = 0, *c* = 1. The phase difference of the gate B changes non-adiabatically for *a* = 1, *b* = 0, *c* = 1 in the normal process.

In this section, we discuss the relationship between logical reversibility and energy dissipation using logically irreversible circuits. Figure 6 shows the schematic of the 1-bit-erasure gate, which is a logically irreversible circuit. The inputs, *a* and *c*, are copied to the outputs, *x* and *z*, respectively. The output, *y*, takes the majority vote of the inputs, *a*, *b*, and *c*. Therefore, it is not possible to determine the input, *b*, from the outputs, and 1-bit information is erased at every logic operation. In the similar way to last section, we investigate the evolution of phase differences for both normal and time-reversal processes. Figure 7 shows the evolution of phase differences of the Josephson junctions in the 1-bit-erasure gate for both normal and time-reversal processes. Depending on the input data combination, the logic states of the gates are not always the same between normal and time-reversal processes due to logical irreversibility. When *a* = 1, *b* = 0, and *c* = 1, it is clear that the circuit is not time reversible and the gate B shows non-adiabatic state change.

**Figure 8:**
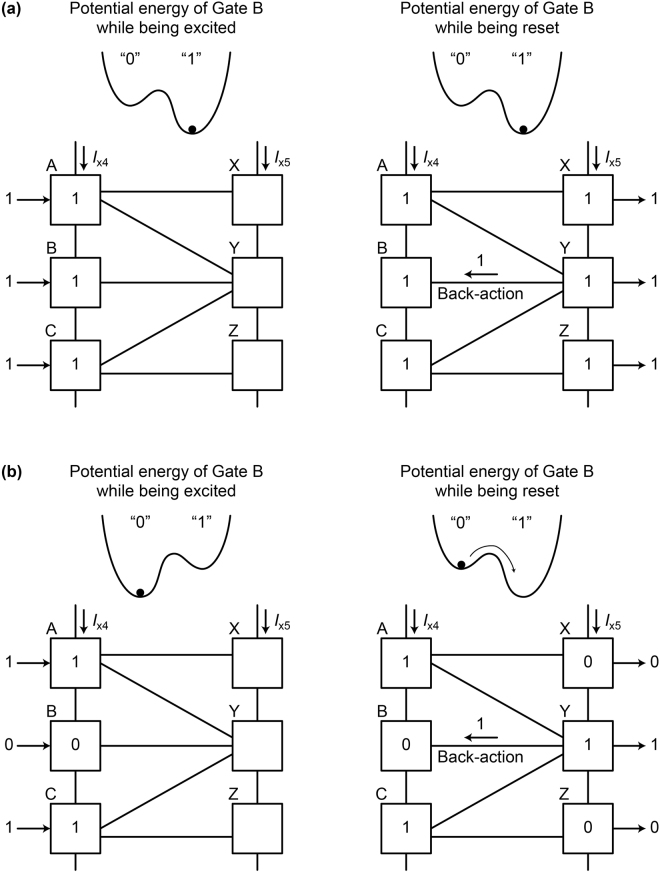
Back-actions in the 1-bit-erasure gate. (**a**) *a* = 1, *b* = 1, *c* = 1. While the gate B is being excited, the potential energy is tilted toward logic 1 due to the input *b*. Likewise, the potential energy of the gate B is tilted toward logic 1 by the back-action from the gate Y while being reset. (**b**) *a* = 1, *b* = 0, *c* = 1. While the gate B is being excited, the potential energy is tilted toward logic 0 due to the input *b*. On the other hand, the potential energy of the gate B is tilted toward logic 1 by the back-action from the gate Y while being reset, which induces a non-adiabatic state change from logic 0 to 1 before the shape of the potential energy returns to a single well.

The reason for the non-adiabatic change can be explained well by Likharev’s argument^23^. Since AQFP gates are magnetically coupled to each other, there always exist interactions between neighboring gates. For example, in Fig. 6, the gate B is coupled to the gate Y, therefore the evolution of potential energy of the gate B is affected by the back-action from the gate Y. According to Likharev’s argument, when a pair of gates, that are coupled to each other, hold different logical values, the state of a gate changes non-adiabatically while the potential energy is being reset from a double-well shape to a single-well shape. Figure 8 explains back-actions in the 1-bit-erasure gate. Figure 8a shows back-actions for *a* = *b* = *c* = 1. During excitation, the potential energy of the gate B is tilted toward logic 1 by the input *b,* and thus the gate B switches to logic 1. While the gate B is being reset, the potential energy is also tilted toward logic 1 by the back-action from the gate Y. Therefore, the gate B is always in the minimum potential energy and phase differences can change adiabatically. Figure 8b shows back-actions for *a* = 1, *b* = 0, *c* = 1. The gate B switches to logic 0, because the input *b* tilts the potential energy toward logic 0 during excitation. On the other hand, while the gate B is being reset, the back-action from the gate Y in logic 1 tilts the potential energy of the gate B toward logic 1. As a result, before the shape of the potential energy returns to a single well, the gate B experiences a non-adiabatic state change from logic 0 to 1, as shown in the figure, which corresponds to the non-adiabatic change of phase differences shown in Fig. 7b. By way of comparison, here we observe the interactions in the RQFP gate. As shown in Fig. 1, the gate B is coupled to the gates X, Y, and Z, the back-actions from which affect the evolution of potential energy of the gate B. In Fig. 4(b), for example, the logic state of the gate B is 0 and the majority of the back-actions from the gates X, Y, and Z is also 0. Therefore, the phase differences in the gate B change adiabatically. For all the AQFP gates in the RQFP gate, interactions work in this way for all the input data combinations, which results in reversible computing with energy dissipation smaller than the Landauer bound, as shown in Fig. 5.

**Figure 9:**
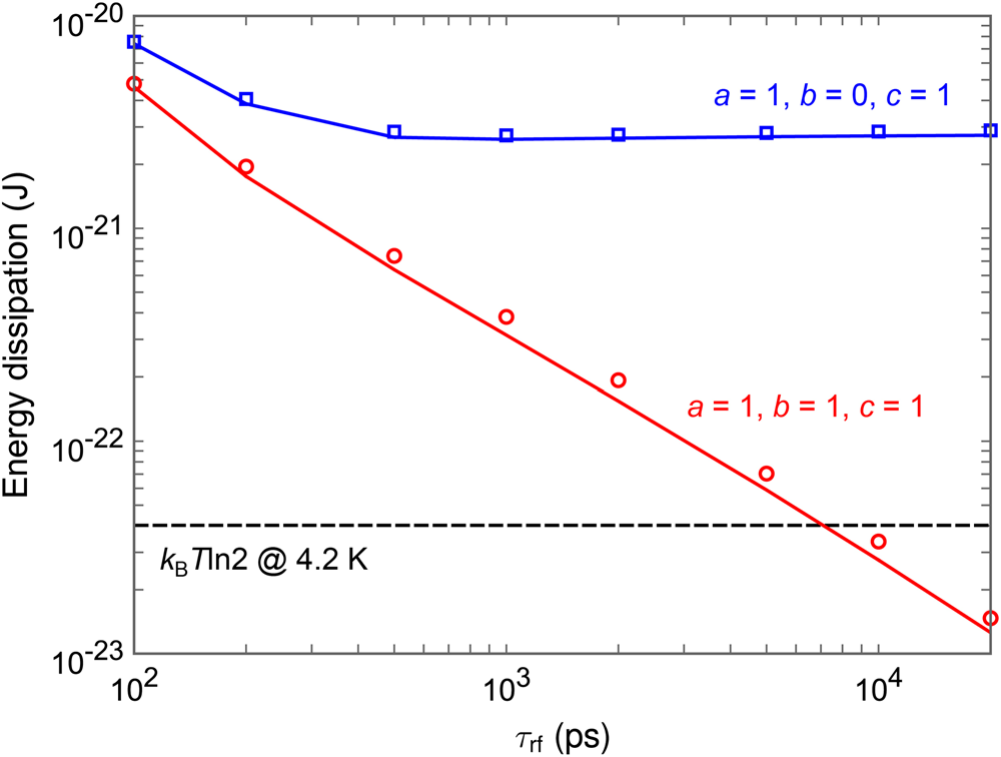
Simulation results of the energy dissipation per logic operation of the 1-bit-erasure gate as a function of the rise and fall time of excitation currents, τ_rf_. The lines show the results without taking into account thermal noise, and the markers show the averaged results over 500 iterations with thermal noise at 4.2 K. For *a* = 1, *b* = 0, *c* = 1, the energy dissipation almost does not depend on τ_rf_.

Figure 9 shows the simulated energy dissipation per logic operation of the 1-bit-erasure gate as a function of τ_rf_, where the lines show the calculation results without taking into account thermal noise and the markers show the averaged values over 500 iterations with 4.2 K thermal noise. While the energy dissipation reduces linearly as τ_rf_ increases for *a* = 1, *b* = 1, and *c* = 1, energy dissipation is almost constant for *a* = 1, *b* = 0, and *c* = 1. This is because, as shown in Fig. 7(b), the gate B experiences non-adiabatic processes and generates heat much larger than the Landauer bound. The above results indicate that, in logically irreversible circuits, interactions between gates induce heat so as to compensate for the reduction in logical entropy due to logical irreversibility.

### Physical Reversibility

**Figure 10:**
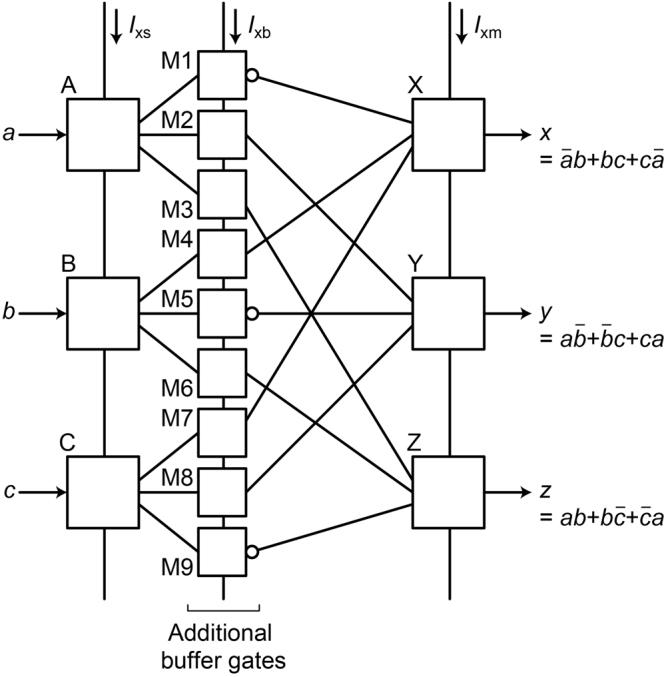
Schematic of the quasi-RQFP gate, which is based on the RQFP gate. Additional buffer gates are added between the SPL and MAJ gates. The quasi-RQFP gate performs the same logic operations as the RQFP gate does. Therefore, the quasi-RQFP gate is logically reversible.

**Figure 11:**
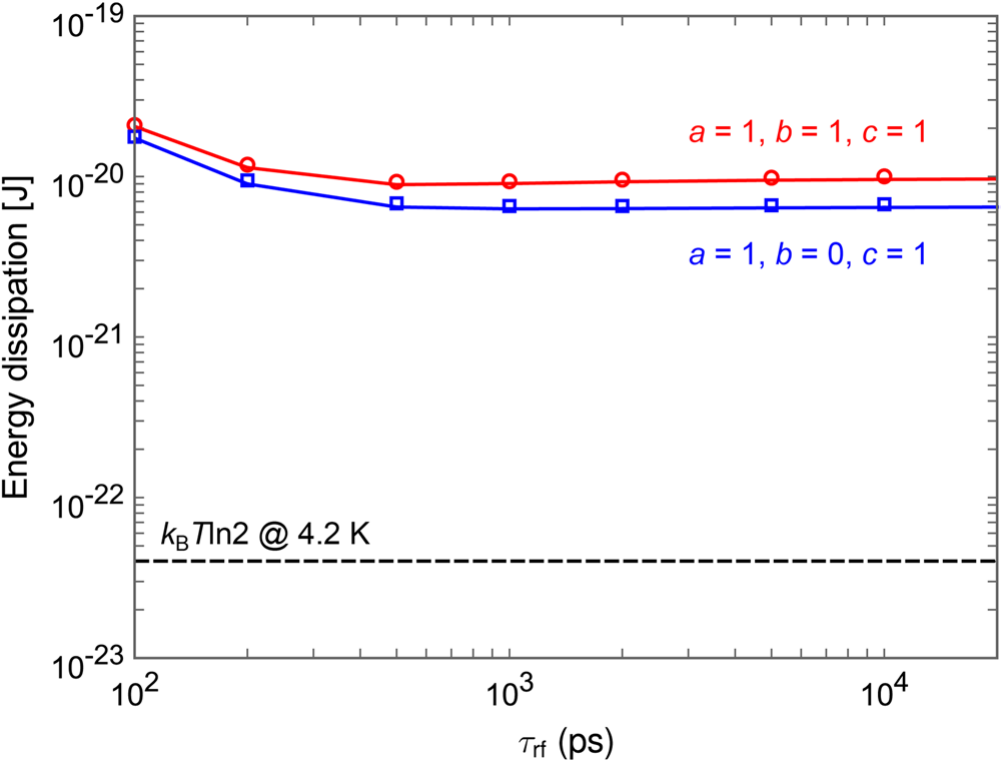
Simulation results of the energy dissipation per logic operation of the quasi-RQFP gate as a function of the rise and fall time of excitation currents, τ_rf_. The lines show the results without taking into account thermal noise, and the markers show the averaged results over 500 iterations with thermal noise at 4.2 K. For all the input data combinations, the energy dissipation almost does not depend on τ_rf_.

**Figure 12:**
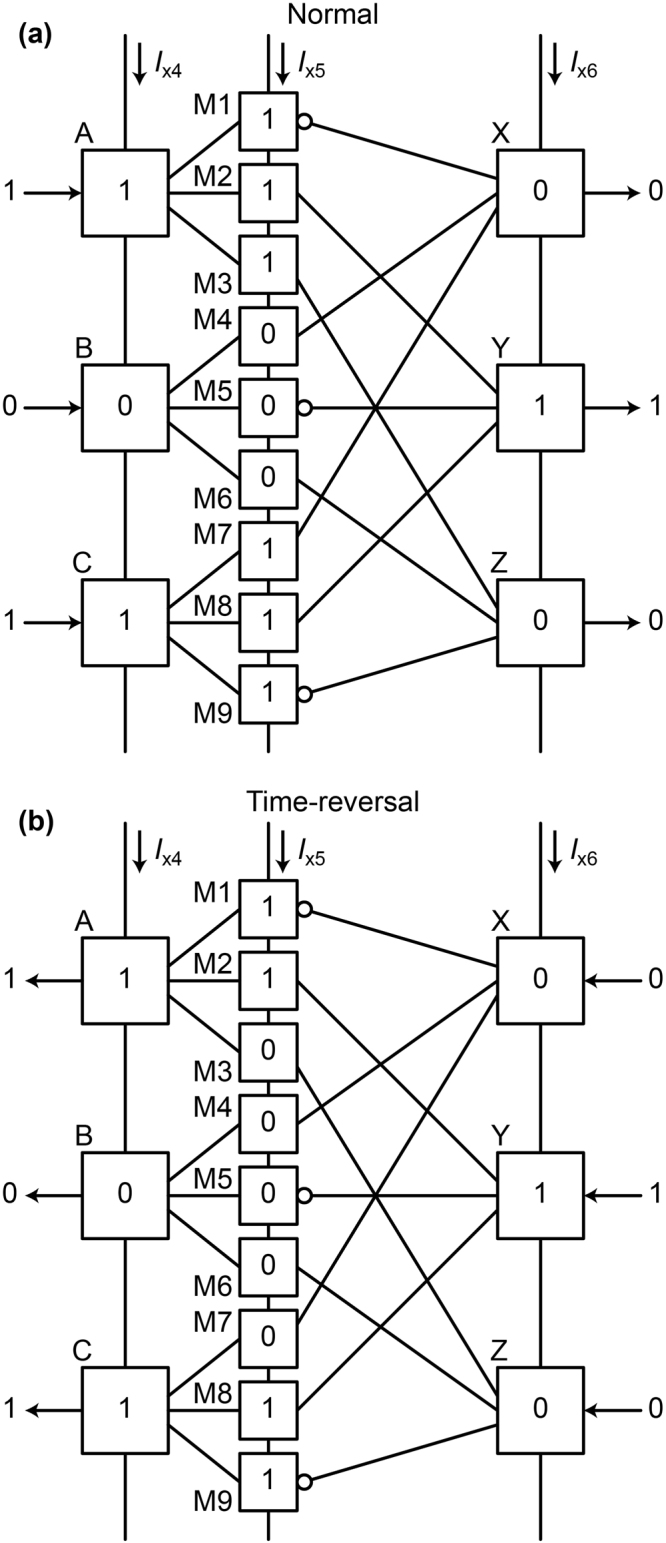
Logic states of the AQFP gates in the quasi-RQFP gate for *a* = 1, *b* = 0, *c* = 1. (**a**) Normal process. (**b**) Time-reversal processes. The logic states of the gates M3 and M7 are not the same between normal and time-reversal processes.

Next, we discuss the relationship between physical reversibility and energy dissipation using physically irreversible circuits. Figure 10 shows the schematic of the quasi-RQFP gate, which is a physically irreversible circuit based on the RQFP gate. Buffer gates are added between SPL and MAJ gates so as to make the gate physically irreversible. The quasi-RQFP gate performs the same logic operations as the RQFP gate, and also data can propagate bi-directionally. Therefore, this circuit is logically reversible. Figure 11 shows the simulated energy dissipation per logic operation of the quasi-RQFP gate as a function of τ_rf_, where the lines show the calculation results without taking into account thermal noise and the markers show the averaged values over 500 iterations with 4.2 K thermal noise. For all the input data combinations, the energy dissipation is almost constant, which indicates that some of the AQFP gates in the quasi-RQFP gate experience non-adiabatic processes. Figure 12 shows the logic states of the AQFP gates in the quasi-RQFP gate for *a* = 1, *b* = 0, and *c* = 1. Unlike the RQFP, the logic states of some AQFP gates are different between normal and time-reversal processes. Therefore, the quasi-RQFP gate is physically irreversible, where the evolution of phase differences is not time reversible. In Fig. 12(a), while the logic state of the gate labeled as M3 is 1, that of the gate Z is 0. As discussed earlier, the back-action from the gate Z biases the potential energy of the gate M3, inducing a non-adiabatic process. In the similar way, the gate M7 also experiences a non-adiabatic process due to the back-action from the gate X. This indicates that, even if the circuit is logically reversible, interactions between gates induce non-adiabatic processes and heat generation in physically irreversible circuits.

Here we discuss the difference in minimum energy bounds between the 1-bit-erasure gate and the quasi-RQFP gate. The energy bounds are determined by the amplitude of back-action currents from neighboring gates and the number of AQFP gates, which experience non-adiabatic processes due to the back-actions. In this study, the circuit parameters of AQFP gates are the same in all the circuits, and thus the amplitudes of back-action currents are considered to be the same between the 1-bit-erasure gate and the quasi-RQFP gate. Therefore, the difference in the energy bounds comes from the difference in the number of AQFP gates, which experience non-adiabatic processes. For the 1-bit-erasure gate with *a* = 1, *b* = 0, c = 1, only the gate B experiences non-adiabatic processes, which gives an energy bound of approximately 3 × 10^−21^ J, as shown in Fig. 9. For the quasi-RQFP gate with *a* = 1, *b* = 0, *c* = 1, the gates M3 and M7 experience non-adiabatic processes, so that the energy bound is approximately 6 × 10^−21^ J, as shown in Fig. 11, which is almost twice as large as that of the 1-bit-erasure gate with *a* = 1, *b* = 0, *c* = 1. For the quasi-RQFP gate with *a* = 1, *b* = 1, *c* = 1, even more gates (M1, M5, and M9) experience non-adiabatic processes, and the energy bound further increases to approximately 1 × 10^−20^ J. Currently, it is not clear how much the back-actions can be reduced and how small energy bounds can be obtained in irreversible gates. Future studies will be required to make more clear the relationship between back-actions and energy dissipation.

## Conclusions

We showed that the RQFP gate is physically reversible by observing the evolution of the phase differences of Josephson junctions. We numerically demonstrated that the energy dissipation per logic operation of the RQFP gate can fall below the Landauer bound. Next, we observed the evolution of phase differences in the 1-bit-erasure gate, which is a logically irreversible circuit. We showed that interactions between gates in the 1-bit-erasure gate induce non-adiabatic process, generating heat so as to compensate for the reduction in logical entropy. We also discussed the relationship between physical reversibility and energy dissipation using the quasi-RQFP gate, which is a physically irreversible circuit based on the RQFP gate. We showed that the quasi-RQFP gate generates heat due to physical irreversibility. The above results show that reversible computing is possible in logically and physically reversible AQFP gates. It is noteworthy that, if the interaction between gates (back-action) is a sole factor causing minimum energy bounds and logical reversibility is tied to physical reversibility as Landauer predicted, logical and physical reversibility is a necessary and sufficient condition for back-action-free operation, and vice versa. This is because, if a system is physically reversible, minimum energy bounds do not appear, and vice versa. So far, we have not discovered any other factor causing minimum energy bounds, and Landauer’s principle has been considered reasonable.

## Methods

### Calculation of energy dissipation

The energy dissipation per logic operation of the RQFP gate, *E*_diss_, was calculated as follows:

$${E}_{{\rm{diss}}}={\int }_{{\tau }_{1}}^{{\tau }_{2}}\{{I}_{{\rm{x}}4}\cdot {v}_{{\rm{x}}4}+{I}_{{\rm{x}}5}\cdot {v}_{{\rm{x}}5}\}dt,$$

where *I*_x4_ and *I*_x5_ are the excitation currents to drive the RQFP gate, *v*_x4_ and *v*_x5_ are the voltages across the current sources of *I*_x4_ and *I*_x5_, respectively, τ_1_ is the time when *I*_x4_ starts to rise, and τ_2_ is the time when *I*_x5_ returns to zero. The markers in Fig. 5 shows the average of *E*_diss_ over 500 iterations at 4.2 K. The energy dissipation of the 1-bit-erasure gate and the quasi-RQFP gate were also calculated in the similar way.
